# Supplementary material for: Design, Synthesis, and Biological Evaluation of N-Acylhydrazones and Their Activity Against Leishmania amazonensis Promastigotes
Source: Microorganisms. 2025 Jul 2;13(7):1563. doi: 10.3390/microorganisms13071563 (PMC12298860; doi:10.3390/microorganisms13071563)
Supplement: Supplementary file 1 [file microorganisms-13-01563-s001.zip › RMN amostras-20241114T155656Z-001.zip!/RMN amostras/composto 13 - PMM 103-17 1Ha.pdf]

PMM 103-17 - Joao Mafra
Op. Eliane

farmanguinhos
Instituto de Tecnologia em Fármacos

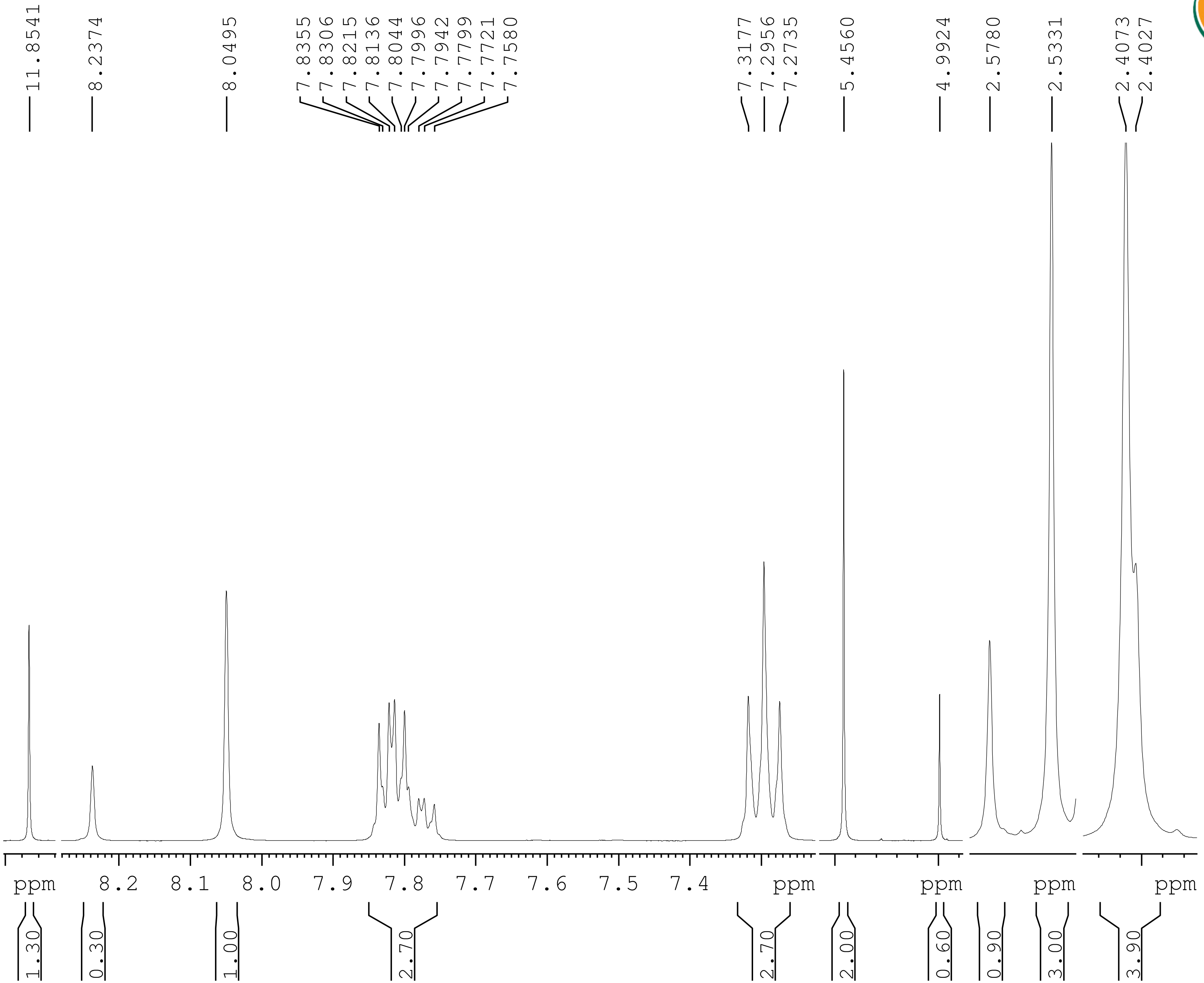


Current Data Parameters
NAME si105181995
EXPNO 10
PROCNO 1

F2 - Acquisition Parameters
Date_ 20180503
Time 11.39 h
INSTRUM spect
PROBHD Z122623_0041 (
PULPROG zg30
TD 65536
SOLVENT DMSO
NS 32
DS 2
SWH 8012.820 Hz
FIDRES 0.244532 Hz
AQ 4.0894465 sec
RG 206.8
DW 62.400 usec
DE 10.00 usec
TE 298.0 K
D1 1.00000000 sec
TD0 1
SFO1 400.1524711 MHz
NUC1 1H
P1 11.50 usec
PLW1 7.30000019 W

F2 - Processing parameters
SI 65536
SF 400.1500017 MHz
WDW EM
SSB 0
LB 0.30 Hz
GB 0
PC 1.00
